# Semantic Cardiac Segmentation in Chest CT Images Using K-Means Clustering and the Mathematical Morphology Method

**DOI:** 10.3390/s21082675

**Published:** 2021-04-10

**Authors:** Beanbonyka Rim, Sungjin Lee, Ahyoung Lee, Hyo-Wook Gil, Min Hong

**Affiliations:** 1 Department of Software Convergence, Soonchunhyang University, Asan 31538, Korea; rim.beanbonyka@sch.ac.kr (B.R.); fijianwa@gmail.com (S.L.); 2 Department of Computer Science, Kennesaw State University, Marietta, GA 30144, USA; alee146@kennesaw.edu; 3 Department of Internal Medicine, Soonchunhyang University Cheonan Hospital, Cheonan 31151, Korea; hwgil@schmc.ac.kr; 4 Department of Computer Software Engineering, Soonchunhyang University, Asan 31538, Korea

**Keywords:** whole cardiac segmentation, chest CT scans, image processing, K-Means clustering, silhouette score, mathematical morphology method

## Abstract

Whole cardiac segmentation in chest CT images is important to identify functional abnormalities that occur in cardiovascular diseases, such as coronary artery disease (CAD) detection. However, manual efforts are time-consuming and labor intensive. Additionally, labeling the ground truth for cardiac segmentation requires the extensive manual annotation of images by the radiologist. Due to the difficulty in obtaining the annotated data and the required expertise as an annotator, an unsupervised approach is proposed. In this paper, we introduce a semantic whole-heart segmentation combining K-Means clustering as a threshold criterion of the mean-thresholding method and mathematical morphology method as a threshold shifting enhancer. The experiment was conducted on 500 subjects in two cases: (1) 56 slices per volume containing full heart scans, and (2) 30 slices per volume containing about half of the top of heart scans before the liver appears. In both cases, the results showed an average silhouette score of the K-Means method of 0.4130. Additionally, the experiment on 56 slices per volume achieved an overall accuracy (OA) and mean intersection over union (mIoU) of 34.90% and 41.26%, respectively, while the performance for the first 30 slices per volume achieved an OA and mIoU of 55.10% and 71.46%, respectively.

## 1. Introduction

Cardiovascular disease (CVD) has been reported as one of the leading causes of death globally and occurs due to functional abnormalities in the heart and blood vessels [1]. In 2016, according to the World Health Organization (WHO), about 17.9 million people died from CVDs, which is equivalent to 31% of all global deaths (mainly from stroke and heart attack) [1]. For instance, one of the CVDs, coronary artery disease (CAD) is a group of abnormalities in blood vessels supplying the heart muscle [1]. CAD is caused by a surplus of calcium in the coronary artery trees. Excessive calcium can narrow the arteries and increase the risk of heart attack [2,3]. Therefore, an early assessment and diagnosis can significantly reduce the life-threatening nature of this CVD and improve quality of life for the afflicted patients [2,3]. In modern medical imaging modalities, computed tomography (CT), magnetic resonance imaging (MRI), and ultrasound are used to assist in identifying abnormal findings in the human body for early assessment and diagnosis [4,5,6,7]. Recently, the non-gated and non-invasive chest CT has been used to provide potential support for investigative imaging tests to interpret cardiac function states [8,9,10]. More detailed characteristics of chest CT images are described in Appendix A.

Cardiac segmentation in chest CT images has played a key role in partitioning the whole chest CT image into a number of anatomically meaningful regions, known as regions of interest (ROIs). Typically, the anatomical ROIs for cardiac segmentation focus on the four chambers of the heart—the left ventricle (LV), right ventricle (RV), left atrium (LA) and right atrium (RA)—or the entire heart anatomy, including the four chambers, coronary arteries and descending thoracic aorta (DA) [8,9,10]. The manual process of cardiac segmentation can be time-consuming and labor intensive for radiologists. To overcome this burden, fully automatic methods have been proposed by applying computer-aided technologies [1,7,8,9,10]. These techniques have been built based on earlier approaches [8] such as graph-based segmenting [11,12], mean-thresholding [13,14,15,16,17] and fuzzy clustering methods [18,19,20,21]. Later, the deep learning approach showed promising successful performance [7,9,10]. The deep learning approach has two learning manners, including supervised learning [22,23,24,25,26], which requires a ground truth to align the loss function, and unsupervised learning [27,28,29], which learns the features without ground truth labeling.

Labeling the ground truth for cardiac segmentation requires the extensive manual annotation of images by the radiologist, which can be time-consuming and labor intensive [7,8,9,10,22,23,24,25,26]. Due to the difficulty in obtaining annotated data and the required expertise as an annotator, an unsupervised approach has been considered in this study. The goal of cardiac segmentation is to partition the whole chest CT image into cardiac anatomical ROIs, with respect to not only the dissimilarity of each pixel’s value, but also the meaningful structure (i.e., geometrical position) of cardiac anatomical ROIs. For example, the Hounsfield units (the quantitative scale of chest CT images) of heart muscles and other muscles in the body are in the same range (almost identical). Detailed information of the Hounsfield unit (HU) of chest CT images is described in Appendix B. Additionally, the cardiac anatomical substructures (i.e., the four chambers, DA and coronary artery) are usually formed in one shape that is mostly arranged in the center of a chest CT image [3,30].

Inspired by the performance of the mean-thresholding method [13,14,15,16,17], we adapted it for this current study by utilizing the K-Means clustering method as a threshold criterion. The K-Means clustering method [31,32] is a simple unsupervised method, which exploits Euclidean distances to compute the mean of all given pixels and assigns pixels into k different clusters based on the nearest mean. However, the anatomical structure of cardiac tissues and the quantitative scale (i.e., HU) of chest CT images are very complicated for cardiac segmentation when using the K-Means clustering method directly. Therefore, we exploited a mathematical morphology method [33,34] to enhance the shifting of the mean threshold. In this paper, semantic whole-heart segmentation combining K-Means clustering as a threshold criterion of mean-thresholding and the mathematical morphology method as a threshold shifting enhancer is proposed. In the proposed approach, the K-Means method is utilized to automatically cluster pixels, while the mathematical morphology method is used to remove noise, fill holes and convex hull; also, prior knowledge of chest anatomical structures [3,30] is required to assist in the awareness of geometrical positions. The silhouette scoring method [32,35] is applied to evaluate the performance of K-Means clustering, while overall accuracy (OA) and mean intersection over union (mIoU) are calculated to evaluate the overall performance of cardiac segmentation.

The remaining parts of the paper are organized as follows. Section 2 describes related works of earlier approaches, such as graph-based, mean-threshold and fuzzy clustering, in addition to deep learning approaches such as supervised and unsupervised learning methods. Section 3 describes a step-by-step hierarchical flow of the proposed methodology. Experimental results and comparison discussions are analyzed in Section 4 and Section 5, respectively. Section 6 concludes our study. Finally, supplemental literature reviews of CT, HU and algorithm tables are presented in Appendix A, Appendix B and Appendix C, respectively.

## 2. Related Works

Shi et al. [11] conducted natural image segmentation by proposing a normalized cut criterion. The method addressed images as a graph partition problem. The normalized cut was used as a measure of dissimilarity between subgroups of a graph. The eigenvalue was utilized to minimize the criteria of the normalized cut. Pedro et al. [12] conducted natural image segmentation through a pairwise region comparison method. The method also addressed images as a graph partition problem. The greedy decision was used as a measurement criterion.

Dorin et al. [13] conducted natural image segmentation by proposing a recursive mean shift procedure to generate M-estimators. The M-estimators were utilized in detecting the modes of the density. The method produced significant results with a low dimension of the space. Larrey-Ruiz et al. [14] conducted cardiac segmentation in chest CT images using multiple threshold values. The thresholding was calculated by the mean value of statistical local parameters (i.e., pixels are in one slice) and global parameters (i.e., pixels are across all slices of a full volume). Huo et al. [15] conducted weakly unsupervised cardiac segmentation as an initial step for coronary calcium detection. The method contained many unrelated anatomical ROIs, such as the spine, ribs, and muscles. The method was adapted from Liao et al. [16], utilizing a convex hull of lungs as the base parameters. Rim et al. [17] conducted whole-heart segmentation by adapting the cardiac segmentation method of Huo et al. [15] and Liao et al. [16]. The method used an alternative threshold of a convex hull of lungs and a convex hull of ribs as a base parameter. As the alternative threshold was manually defined by the mathematical geometry of the lungs, the method is an ad hoc set of each image.

Radha et al. [18] conducted brain image segmentation using an intelligent fuzzy level set. Quantum particle swarm optimization was proposed to improve the steadiness and meticulousness in order to reduce opening sensitivity. The results for this method showed that it could optimize up to 15% more than the original fuzzy level set method. However, the method experienced challenges with neoplastic or degenerative ailment images. Versaci et al. [19] conducted image edge detection by proposing a new approach of fuzzy divergence and fuzzy entropy. The proposed fuzzy entropy used fuzzy divergence as the distances between fuzzified images, which were computed by means of fuzzy divergence. Chanda et al. [20] conducted cardiac MRI image segmentation using a fuzzy-based approach. The method was based on morphological, threshold-based segmentation and fuzzy-based edge detection. The method achieved more than 90% accuracy. Kong et al. [21] conducted an image segmentation method using an intuitionistic fuzzy C-Means clustering algorithm. The method defined a modified non-membership function, an initial clustering center based on grayscale features, an improved nonlinear kernel function and a new measurement of fuzzy entropy. The method outperformed existing algorithms, but it took a lot of computational time.

Ronneberger et al. [22] conducted an electron microscopy (EM) image segmentation using supervised deep learning by defining U-net architecture. The network consisted of 23 convolutional layers. The experiment was trained with 30 images of size 512 × 512 and received an average IOU of 92%. The U-net model has become the gold standard for biomedical image segmentation. Payer et al. [23] conducted whole-heart segmentation using supervised deep learning by defining two-step CNN architecture. The experiment was trained on CT images with 20 volumes of size 300 × 300 × 188 and achieved an average dice similarity coefficient of 90.8%. Ahmed et al. [24] conducted whole-heart segmentation using supervised deep learning by defining a CNN network and using stacked denoising auto-encoders. The experiment was trained on CT images of eight subjects and achieved an average accuracy of 93.77%. Liao et al. [25] conducted 3D whole heart segmentation using supervised deep learning by defining a multi-modality (i.e., CT and MRI) transfer learning network with adversarial training. The network introduced the attention mechanism into the U-net network. The experiment was trained on 60 CT volumes with a dice value of 0.914. Max et al. [26] conducted multi-modality (i.e., CT and MRI) 3D whole-heart segmentation using supervised deep learning by defining a shape encoder–decoder network. The experiment was trained on 15 CT volumes with a dice value of 0.653.

Xia et al. [27] conducted natural image segmentation using unsupervised deep learning by defining a W-net architecture. The network was a concatenation of two U-net networks. The experiment was trained on 11,530 images and achieved a probabilistic rand index (PRI) of 0.86. Joyce et al. [28] conducted multi-class medical image segmentation on Myocardium (MYO), left ventricle (LV) and right ventricle (RV) regions using unsupervised deep learning by defining a generative adversarial network (GAN) model. The experiment was trained on 20 CT volumes with a dice value of 0.51. Perone et al. [29] conducted medical image segmentation using unsupervised deep learning by defining a self-ensembling architecture. The experiment was trained on MRI images from 100 subjects and achieved the best dice value of 0.847.

## 3. Methodology

### 3.1. Overall Workflow of the Proposed Method

**Figure 1:**
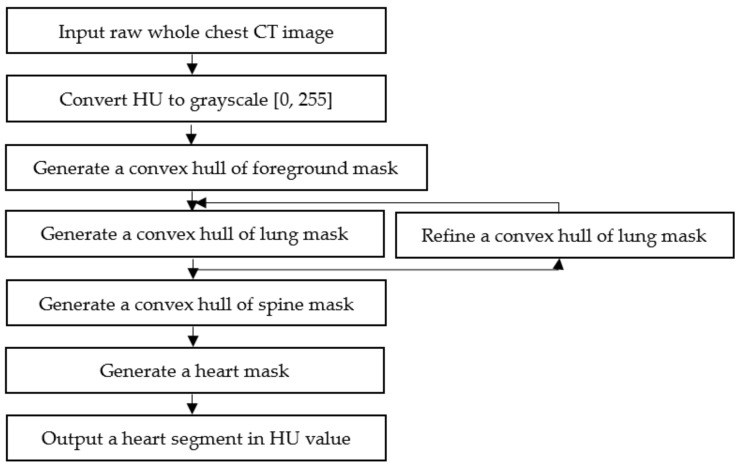
Overall workflow of the step-by-step hierarchical cardiac segmentation process using K-Means clustering and mathematical morphology method. Abbreviations: CT, computed tomography; HU, hounsfield unit.

**Figure 2:**
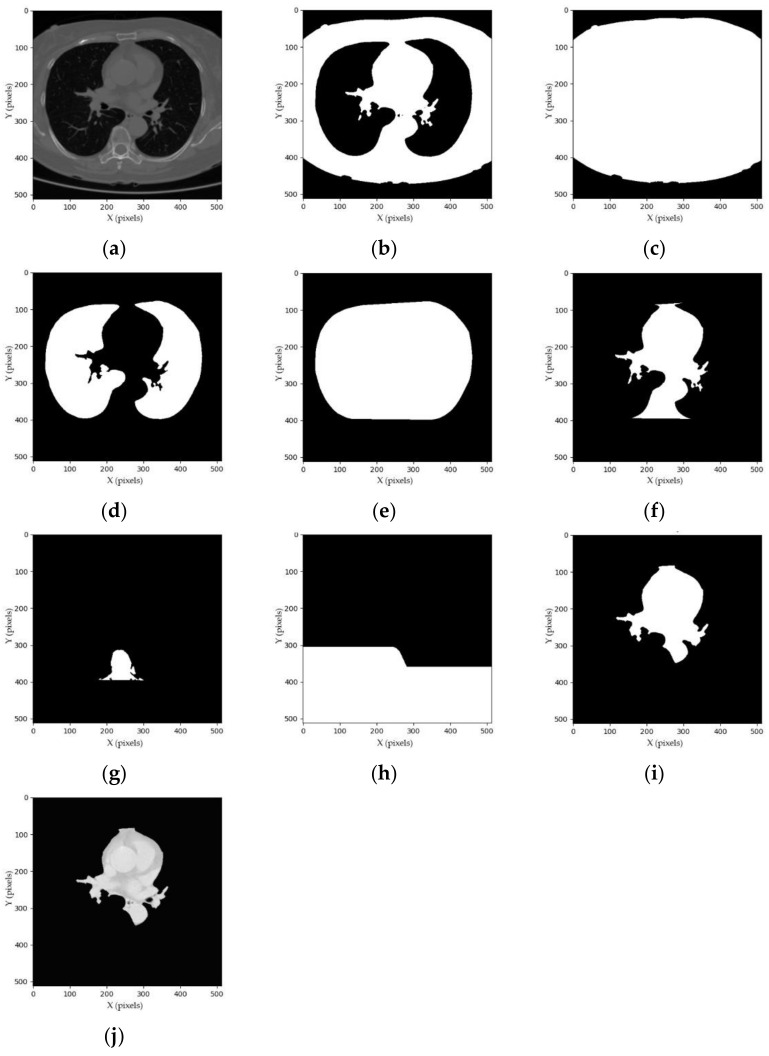
Examples of the step-by-step hierarchical process: (**a**) whole chest CT image; (**b**) foreground mask; (**c**) convex hull of foreground mask; (**d**) lung mask; (**e**) convex hull of lung mask; (**f**) intermediate heart mask; (**g**) spine mask; (**h**) convex hull of spine mask; (**i**) heart mask; and (**j**) segmented heart image.

**Figure 3:**
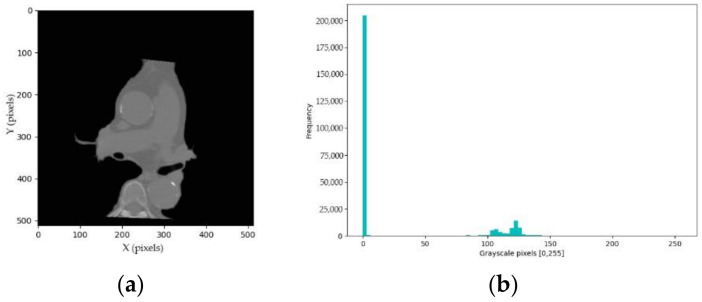
Examples of intermediate heart segmentation: (**a**) intermediate heart image; and (**b**) corresponded frequency histogram.

The segmentation process is intended to generate a binary mask of whole-heart anatomical ROIs, including the four chambers, coronary arteries, and DA, as shown in Figure 1, Figure 2 and Figure 3 and listed in Appendix C. As the cardiac anatomical ROIs are formed in one shape, arranged mostly in the center of a chest CT image [3,30], a step-by-step hierarchical process to enhance the mean-threshold method from the corner towards the center region of the chest CT image is proposed. Firstly, the air substance at the corner of a chest CT image is filtered by a convex hull of foreground mask. Then, other human body substances such as fat, muscles and lungs are filtered by a convex hull of lung mask. Finally, the spine substance is filtered by a convex hull of spine mask. More details of each step are explained in the following sub-sections.

### 3.2. Grayscale Conversion

Firstly, the raw CT image is read in HU pixels, which is the standard radio-density scale. One CT image has a resolution of 512×512×1 (i.e., width × height × channels), with the range around [−1000, +1000]. In the current study, 56 images per volume are trimmed. Thus, one CT volume is 512×512×56 in size. The HU pixels are converted into grayscale pixels using the standard range normalization, as shown in Equation (1).

(1) $$X=\;255\times \frac{Y-Y_{min}}{Y_{max}-Y_{min}}$$

where Y is a set of HU pixels, $Y\subset \;{\left[-1000,+1000\right]}^{3D}$, and X is a set of grayscale pixels, $X\subset \;{\left[0,255\right]}^{3D}$ and 3D=512×512×56. $Y_{min}$ and $Y_{max}$ are the minimum and maximum value of HU pixels, respectively. An example of a chest CT image in grayscale pixels is shown in Figure 2a.

### 3.3. Convex Hull of Foreground Mask

The K-Means method (known as the unsupervised technique in clustering literature [31,32]) is used to cluster the whole chest CT image into foreground (i.e., anatomical ROIs such as fat, muscle, lungs, heart and other) and background (i.e., air) clusters. Given k=2 clusters, and the set of X grayscale pixels $X\subset \;{\left[0,255\right]}^{2D}$, the k centroid clusters C are calculated by minimizing the function ∅, as shown in Equation (2).

(2) $$\begin{array}{c} c_{i}=\frac{1}{\left|C_{i}\right|}\underset{x\in C_{i}}{\sum }x\\ \emptyset =\underset{x\epsilon X}{\sum }\underset{c\in C}{\min}∥x-c{∥}^{2} \end{array}$$

where the centroid clusters $C=\left\{c_{1},\;c_{2},\;\ldots ,\;c_{k}\right\}$. The foreground binary mask F, $F\subset \;{\left[0,1\right]}^{2D}$ is the result of the thresholding condition, as shown in Equation (3).

(3) $$\begin{array}{c} \tau \;=\;\frac{1}{k}\underset{j\in k}{\sum }C_{j}\\ F=\;\left\{\begin{array}{c} 1\;if\;X>\tau \\ 0\;else \end{array}\right. \end{array}$$

where the foreground threshold τ is calculated by the mean of k centroid clusters C. Lines randomly situated at the bottom of the chest in the CT images were observed, as shown in Figure 2a, which are considered as noise. To remove this noise, the morphological binary opening operation [33,34] φ is conducted with the default structure element from [34]. The operation is iterated twice: $F=\varphi \left(\varphi \left(F\right)\right)$. Then, the morphological convex hull operation [33,34] $F=\omega \left(F\right)$ is adapted to cover all human body substances as foreground, as shown in Equation (4).

(4) $$\begin{array}{c} A_{r}^{i}\;=\;\left(A_{r-1}^{i}\propto B^{i}\right)\;\cup \;F\\ D^{i}=A_{r}^{i}\;if\;A_{r}^{i}is\;equal\;to\;A_{r-1}^{i}\\ \omega \;=\;\cup_{i}^{4}D^{i} \end{array}$$

where F is the foreground mask and $B^{i}$ is the default structure elements with *i* = 1, 2, 3, 4. Given that $A_{0}^{i}$ is a foreground mask ($A_{0}^{i}=F)$, the $A_{r}^{i}$ (where *r* = 1, 2, 3, …) is iteratively applied by a hit-or-miss transform ∝ with $B^{i}$; and when it converges (i.e., $A_{r}^{i}$ is equal to $A_{r-1}^{i}$), it is united with F, which is referred to as $D^{i}$. Then, the convex hull ω is a union of $D^{i}$. The examples and generation processes of the foreground mask and the convex hull of foreground mask are shown in Figure 2b,c and Table A2, respectively.

### 3.4. Convex Hull of Lung Mask

The process of generating a convex hull of lung mask is intended to remove fat, muscle and rib substances. Firstly, the lung mask is computed. The foreground threshold τ is used to compute the lung mask. The enhanced grayscale pixels E, $E\subset \;{\left[0,255\right]}^{2D}$ are computed to assist the thresholding. Then, the lung mask L, $L\subset \;{\left[0,1\right]}^{2D}$ is computed as shown in Equation (5).

(5) $$\begin{array}{c} E=\;\left\{\begin{array}{c} 255\;if\;F==0\\ X\;else\; \end{array}\right.\\ L=\;\left\{\begin{array}{c} 1\;if\;E<\tau \\ 0\;else \end{array}\right. \end{array}$$

There are blood vessels within the lungs, which result in many small holes in lung mask L. To fill those small holes, the morphological binary closing operation [33,34] θ is conducted with the default structure element from [34]. The operation is iterated twice: $L=\theta \left(\theta \left(L\right)\right)$. Then, the convex hull of lung mask U, $U\subset \;{\left[0,1\right]}^{2D}$ is computed by the morphological convex hull operation [33,34] ω adapted from Equation (4), as shown in Equation (6).

(6) $$U=\omega \left(L\right)$$

The intermediate heart mask I, $I\subset \;{\left[0,1\right]}^{2D},$ is a bitwise AND operation between the convex hull of lung mask U and an inversion of lung mask L, which is computed as shown in Equation (7).

(7) $$I=U\;\&\;\sigma \left(L\right)$$

where $\sigma \left(L\right)$ is a bitwise NOT operator: $\sigma \left(L\right)=NOT\left(L\right)$. The examples and generation processes of the lung mask, convex hull of lung mask and intermediate heart mask are shown in Figure 2d–f and Table A3, respectively.

### 3.5. Convex Hull of Spine Mask

The process of generating a convex hull of spine mask is intended to remove spine pixels and other substances under the spine and DA. Firstly, the spine mask is computed. The enhanced intermediate heart grayscale pixels E, $E\subset \;{\left[0,255\right]}^{2D}$ are computed to assist the thresholding, as shown in Figure 3a. Then, the k centroid clusters C of the K-Means method adapted from Equation (2) are computed to divide the enhanced grayscale pixels E into background, heart and spine clusters with a value of k=3. The spine pixels are brighter compared to the background and heart pixels, as shown in Figure 3a. We can assume that spine pixels are in the last right cluster in Figure 3b. Therefore, the spine threshold τ is the maximum centroid of clusters C. Then, the spine binary mask S, $S\subset \;{\left[0,1\right]}^{2D}$, is a result of a thresholding condition, which is defined as shown in Equation (8).

(8) $$\begin{array}{c} E=\;\left\{\begin{array}{c} 0\;if\;I==0\\ X\;else\; \end{array}\right.\\ \tau \;=\;\underset{j\in k}{max}(C_{j})\\ S=\;\left\{\begin{array}{c} 1\;if\;E>\tau \\ 0\;else\; \end{array}\right. \end{array}$$

The morphological binary closing θ and opening operation φ are applied to fill holes and remove islands, respectively. Each operation is iterated twice: $S=\varphi \left(\varphi \left(S\right)\right)$ and then, $S=\theta \left(\theta \left(S\right)\right)$.

The spine mask S does not cover pixels of substances around the spine and under the DA, as shown in Figure 2g. To remove those pixels, the convex hull of spine mask is computed. $\left(x0,y0\right)$ and $\left(xc,yc\right),$ which are denoted as the top and center coordinates of the white convex polygon in the spine mask S, are computed by the region properties function ρ and $\rho^{\prime }$ from [34]: $\left(x0,y0\right)=\;\rho \left(S\right)$ and $\left(xc,yc\right)=\;\rho^{\prime }\left(S\right)$, respectively. Then, the convex hull of spine mask P, $P\subset \;{\left[0,1\right]}^{2D},$ is defined, as shown in Equation (9).

(9) $$\begin{array}{c} P=\;\left\{\begin{array}{c} 1\;if\;S\{y>y0\;and\;x<x0\}\\ S\;else\; \end{array}\right.\\ P=\;\left\{\begin{array}{c} 1\;if\;P\{y^{\prime }>yc\}\\ P\;else \end{array}\right. \end{array}$$

where x and y are a set of coordinates of spine mask S and $y^{\prime }$ is a set of row axes of convex hull spine mask P. Then, the heart mask H, $H\subset \;{\left[0,1\right]}^{2D}$ is a bitwise AND operation between the intermediate heart mask I and an inversion of spine mask convex hull P, which is computed as shown in Equation (10).

(10) $$H=I\;\&\;\sigma \left(P\right)$$

where $\sigma \left(P\right)$ is a bitwise NOT operator: $\sigma \left(P\right)=NOT\left(P\right)$. The examples and generation processes of the spine mask, convex hull of spine mask and heart mask are shown in Figure 2g–i and Table A4, respectively.

### 3.6. Heart Pixel Segmentation

This section explains how to filter the heart image from the heart mask. Given whole chest HU pixels Y, $Y\subset \;{\left[-1000,+1000\right]}^{2D},$ and the heart mask H, $H\subset \;{\left[0,1\right]}^{2D}$, the segmented heart pixels Z, $Z\subset \;{\left[-1000,+1000\right]}^{2D}$ are computed as shown in Equation (11).

(11) $$Z=\;\left\{\begin{array}{c} Y\;if\;H==1\\ -1000\;else \end{array}\right.$$

The examples and generation processes of segmented heart images are shown in Figure 2j and Table A5, respectively.

### 3.7. Convex Hull of Lung Mask Refinement

**Figure 4:**
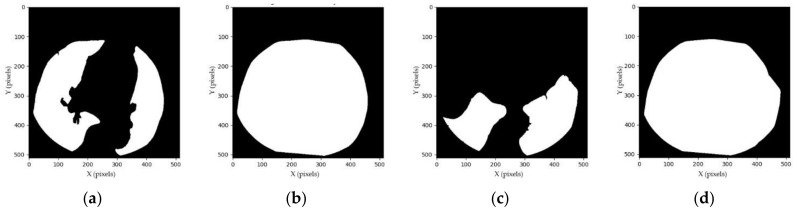
Examples of refining a convex hull of lung mask: (**a**) lung mask of the (n−i)th slice; (**b**) convex hull of the (n−i)th slice; (**c**) lung mask of the nth slice; and (**d**) refined convex hull of lung mask of the nth slice.

The proposed segmentation method depends on the convex hull of lung mask, similar to Huo et al. [14] and Rim et al. [15]. Empirically, when the lungs are not well surrounding the heart region, the convex hull of lung mask fails to compute intermediate heart mask I using Equation (7). In this case, the convex hull of lung mask is refined, as shown in Figure 4. The lungs were observed to vanish little by little from the CT image when the liver appears. Thus, within 56 slices per CT volume, the last lungs-well-surrounded slice is recorded and used as the global parameters for the next slice. Then, the local and global parameters are combined to compute the convex hull of lung mask.

Given N is the number of slices in one volume (i.e., N=56) and y0 is the top row axis of the white convex polygon in the lung mask $L^{n-i}$ and $L^{n}$ , $L\subset \;{\left[0,1\right]}^{3D}$, the convex hull of lung mask U, $U\subset \;{\left[0,1\right]}^{3D}$ is computed by a bitwise OR operation, as shown in Equation (12).

(12) $$U^{n}=\;\left\{\begin{array}{c} U^{n}\left|\;U^{n-i}\;if\;L^{n}\left\{y0\right\}\right.>L^{n-i}\left\{y0\right\}\}\\ U^{n}\;else\; \end{array}\right.$$

where n and (n−i) are the current and the last lungs-well-surrounded slice in N, respectively. The examples and generation processes of the refined convex hull of lung mask are shown in Figure 4 and Table A6, respectively.

## 4. Experimental Results

### 4.1. Experimental Setup

The current experiment was conducted on a Windows 10 computer with an Intel Core™ i7-9700 CPU @ 3.00 GHZ, 32.0 GB RAM, and an NVIDIA GeForce RTX 2070 graphics card. The code was written in Python language with the Scikit image library [34] and the K-Mean method of the Scikit learn library [32]. The method was applied on a dataset from Soonchunhyang University Cheonan Hospital [36]. The dataset was acquired randomly from 500 subjects who were scanned using a CT scanner (Phillips iCT 256) during 2019. The chest CT slices were captured in diverse ranges containing between 56 to 84 slices. The first 56 slices were selected for the current study. The resolution of each slice was the same at 512 × 512 pixels. The FOV was 250 × 250 mm and the slice thickness was 2.5 mm. All data were stored in dicom 3.0 format [37].

### 4.2. Silhouette Score

The silhouette method [32,35] was applied to score how well the K-Mean method [31,32] separated the clusters. The formula of the silhouette method is shown in Equation (13).

(13) $$Score=\frac{b\left\{i\right\}-a\left\{i\right\}}{\max\left(a\left\{i\right\},b\left\{i\right\}\right)}$$

where a is the average distance from the ith pixel to all pixels in the same cluster and b is the average distance from ith pixel to all pixels in the closest cluster. The score has a range of [−1, +1]. If score=−1, it means that the clusters are not well separated. If score=+1, it means that the clusters are well separated. If score=0, it means that the clusters are overlapping.

The evaluation was analyzed on three cases where M denotes the number of images: (1) the first 30 slices from the 1st to 30th slice: M=500×30; (2) the last 36 slices from the 31st to 56th slice: M=500×36; and (3) a full volume per subject from the 1st to 56th slice: M=500×56, as shown in Table 1.

The K-Means method was applied twice for foreground filtering and spine filtering in Equation (2) of Section 3.3 and Equation (8) of Section 3.5, respectively. For foreground filtering, the K-Means method of the three cases achieved mean scores of 0.2891, 0.2904 and 0.2903, respectively. For spine filtering, the K-Means method of the three cases achieved mean scores of 0.5367, 0.5356 and 0.5356, respectively. For overall foreground and spine filtering, the K-Means method for the three cases achieved mean scores of 0.4129, 0.4130 and 0.4130, respectively. We noticed that the K-Means method performed the spine clustering with higher scores than the foreground clustering. Additionally, the K-Means method achieved almost the same mean scores for all three cases in overall foreground and spine filtering.

### 4.3. Human Visual Inspection Evaluation

An intersection over union IoU of each subject, an overall accuracy OA and a mean intersection over union mIoU were calculated for overall segmentation, as shown in Equation (14).

(14) $$\begin{array}{c} IoU=\frac{TP_{n}}{TP_{n}+FP_{n}+FN_{n}}\\ mIoU=\frac{1}{N}\sum IoU_{n}\\ OA=\frac{\sum TP_{n}}{M} \end{array}$$

where N is the number of subjects (i.e., 500 subjects) and n is the nth subject in N. M is the number of images. TP, FP and FN represent number of images of true positives, false positives and false negatives of the segmentation result, respectively. OA and mIoU evaluate the overall quality of the segmentation, and the IoU of each subject evaluates the quality of all images per subject.

**Figure 5:**
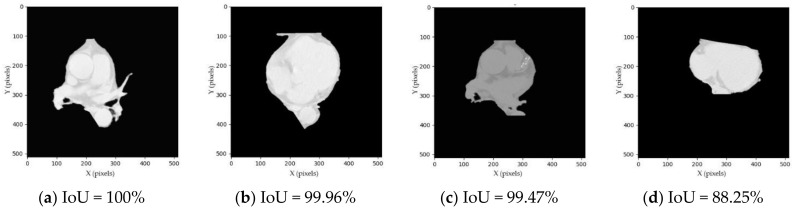

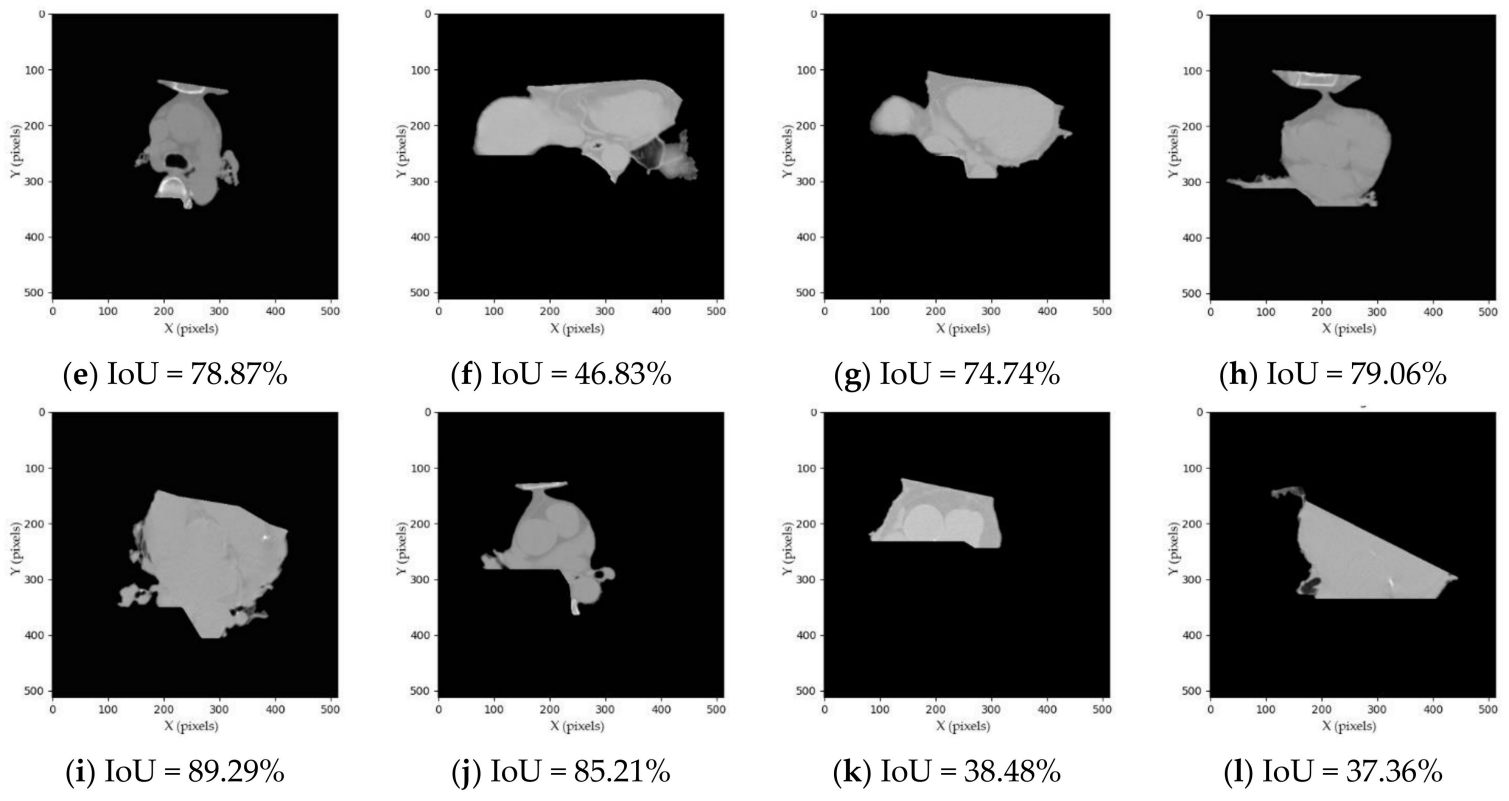
Examples of segmented heart results. (**a**,**b**) TP of whole-heart segmentation; (**c**,**d**) TP of four-chamber segmentation; (**e**,**f**) FP of whole-heart segmentation; (**g**,**h**) FP of four-chamber segmentation; (**i**,**j**) FN of whole-heart segmentation; (**k**,**l**) FN of four-chamber segmentation. Abbreviations: IoU, intersection over union; TP, true positive; FP, false positive; FN, false negative.

Since our segmentation method does not have a ground truth for validating, we conducted a visual inspection manually in which the human error rate was assumed to be around 5%. If the segmented image consists of four chambers, the coronary arteries and the DA, it is considered as a whole-heart segmentation, as shown in Figure 5a,b,e,f,i,j. If the segmented image consists of four chambers and the coronary arteries but misses DA, it is considered as a four-chamber segmentation, as shown in Figure 5c,d,g,h,k,l.

**Figure 6:**
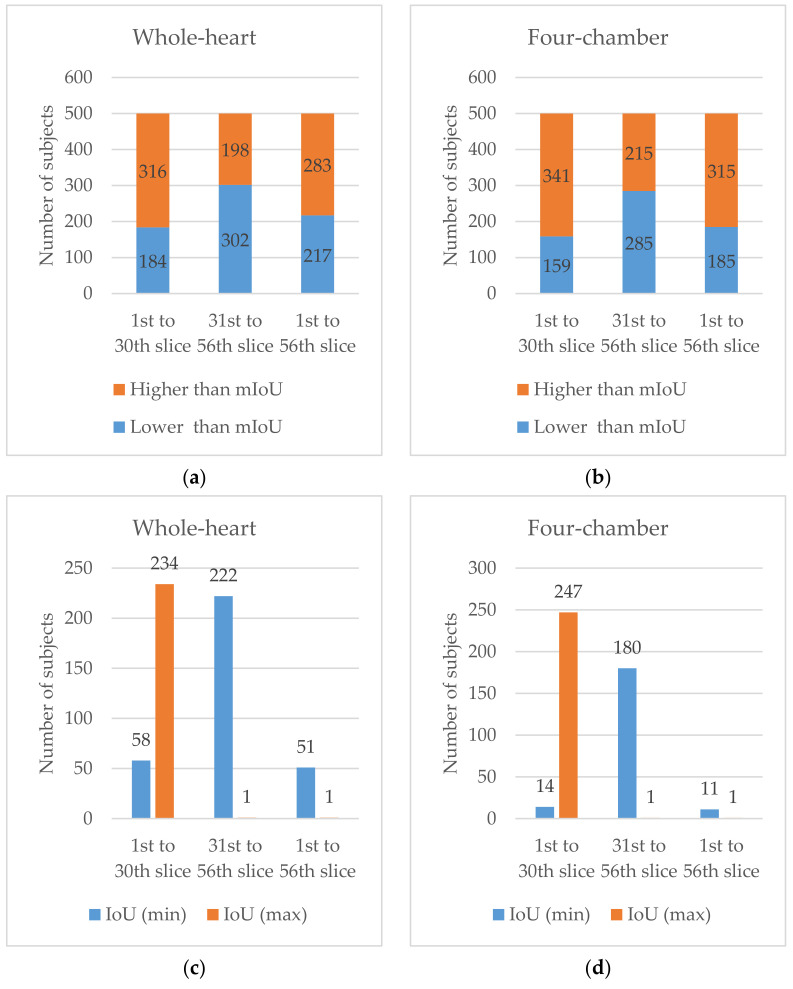
Number of subjects: (**a**) whose IoU was lower than the mIoU (blue) and higher than the mIoU (orange) for whole-heart segmentation; (**b**) whose IoU was lower than the mIoU (blue) and higher than the mIoU (orange) of four-chamber segmentation; (**c**) whose IoU was minimum (blue) and maximum (orange) for whole-heart segmentation; and (**d**) whose IoU was minimum (blue) and maximum (orange) for four-chamber segmentation.

For the first 30 slices, the segmentation achieved high performance on both whole-heart and four-chamber segmentations with OA and mIoU values of 55.10%, 71.46%, 82.62% and 82.62% respectively, as shown in Table 2. Among the 500 subjects, there were 316 and 341 subjects whose IoU was higher than the mIoU for whole-heart and four-chamber segmentations, respectively, as shown in Figure 6a,b. Additionally, the minimum and maximum IoU were 0% and 100%, respectively. Among the 500 subjects, there were 58 and 14 subjects whose IoU was 0%, while there were 234 and 247 subjects whose IoU was 100% for whole-heart and four-chamber segmentations, respectively, as shown in Figure 6c,d.

For the last 36 slices, the segmentation achieved low performance on both whole-heart and four-chamber segmentations with OA and mIoU values of 8.37%, 12.60%, 10.35% and 15.42%, respectively. Among the 500 subjects, there were 198 and 215 subjects whose IoU was higher than the mIoU for whole-heart and four-chamber segmentation, respectively. Additionally, the minimum and maximum IoU are 0% and 69.23%, respectively. Among the 500 subjects, there were 222 and 180 subjects whose IoU was 0%, while there were 1 and 1 subjects whose IoU was 69.23% for whole-heart and four-chamber segmentation, respectively.

For the full volume, the segmentation achieved good performance on both whole-heart and four-chamber segmentations with OA and mIoU values of 34.90%, 41.26%, 50.91% and 54.10%, respectively. Among the 500 subjects, there were 283 and 315 subjects whose IoU was higher than the mIoU for whole-heart and four-chamber segmentation, respectively. Additionally, the minimum and maximum IoU were 0%, 0%, 85.45% and 89.13%, respectively. Among the 500 subjects, there were 51 and 11 subjects whose IoU was 0%, while there were 1 and 1 subjects whose IoU was 85.45% and 89.13% for whole-heart and four-chamber segmentation, respectively.

## 5. Discussion

Among the mean-thresholding methods proposed by [13,14,15,16,17], there is one paper by Larrey-Ruiz et al. [14] in which cardiac segmentation on 32 chest CT images was conducted by defining multiple threshold values. The thresholding was calculated by the mean value of statistical local parameters (i.e., pixels were in one slice) and global parameters (i.e., pixels were across all slices of a whole volume). Table 3 shows comparison results for the top 32 subjects of our proposed method with Larrey-Ruiz et al. [14] for whole-heart segmentation. For the first 30 slices, our results outperform Larrey-Ruiz et al. [14] in both OA and $A\;\left(max\right)$ (maximum accuracy) with values of 100% and 100%, and 94.42% and 99.81%, respectively. For the full volume, Larrey-Ruiz et al. [14] outperforms our method in OA with values of 87.64% and 73.66%, respectively. However, our method outperforms Larrey-Ruiz et al. [14] in $A\;\left(min\right)$ with values of 67.85% and 46.95%, respectively.

Among the unsupervised deep learning approaches proposed by [27,28,29], there is one paper by Joyce et al. [28] in which multi-class medical image segmentation was conducted on MYO, LV and RV regions on 20 CT volumes with a dice value (mDice) of 0.51. For the top 20 subjects, our result of a full volume for whole-heart segmentation outperforms Joyce et al. [28] with an mIoU of 0.7833, as shown in Table 4.

## 6. Conclusions

This paper presented semantic whole-heart segmentation combining K-Means clustering as a threshold criterion of the mean-thresholding method and the mathematical morphology method as a threshold shifting enhancer. The experiment was conducted on 500 subjects in two cases: (1) 56 slices per volume containing full heart scans, and (2) 30 slices per volume containing about half of the top of heart scans before the liver appears. In both cases, the results showed an average silhouette score of the K-Means method, with a value of 0.4130. Additionally, the experiment on 56 slices per volume achieved an OA and mIoU of 34.90% and 41.26%, respectively; while the performance result on the first 30 slices per volume achieved an OA and mIoU of 55.10% and 71.46%, respectively.

High performance was achieved when the heart was well surrounded by lungs. Otherwise, low performance was achieved. The low performance was likely caused by the lack of filtering of the liver, as both the HU pixels and geometrics of the organ could not be used as a criterion for thresholding. Additionally, the goal of the proposed research was to segment the whole heart. However, the results showed that the four-chamber segmentation outperformed the whole-heart segmentation. The outperformance was due to a failure in generating the convex hull of spine mask.

There are limitations in this study, such as the failure of DA segmentation and liver removal, but the main contributions of our proposed method can be summed up as the following: (1) we proposed fully unsupervised semantic whole-heart segmentation from chest CT images; (2) we proposed the K-Means method as a thresholding criterion and the mathematical morphology method as a threshold-shifting enhancer; and (3) we demonstrated good performance for the first 30 slices, which will be able to be used as an initial step for other cardiac applications in other CADs. Finally, the future direction of our research is to conduct an unsupervised deep learning approach to overcome the abovementioned limitations.

## Figures and Tables

**Table 1:** Silhouette scores for 500 subjects. The shilhouette scores of three cases are described in all filtering (foreground, spine, and overall foreground and spine filtering).

	Min	Max	Mean	±SD
**Foreground filtering**				
1st to 30th slice	0.0150	0.4341	0.2891	0.0879
31st to 56th slice	−0.1002	0.5712	0.2904	0.1050
1st to 56th slice	−0.1002	0.5712	0.2903	0.1040
**Spine filtering**				
1st to 30th slice	0.4176	0.6332	0.5367	0.0455
31st to 56th slice	0.3756	0.67359	0.5356	0.0571
1st to 56th slice	0.3756	0.6735	0.5356	0.0565
**Overall Foreground and Spine filtering**				
1st to 30th slice	0.4176	0.5337	0.4129	0.0605
31st to 56th slice	0.1467	0.6053	0.4130	0.0762
1st to 56th slice	0.1467	0.6053	0.4130	0.0753

**Table 2:** Evaluation results of 500 subjects (%). The OA, mIoU, IoU (min) and IoU (max) of three cases are described in both segmentations (whole-heart and four-chamber segmentation). Abbreviations: OA, overall accuracy; mIoU, mean intersection over union; IoU (min), minimum IoU; IoU (max), maximum IoU.

	OA	mIoU	$IoU\;\left(min\right)$	$IoU\;\left(max\right)$
**Whole-heart**				
1st to 30th slice	55.10	71.46	0.00	100.00
31st to 56th slice	8.37	12.60	0.00	69.23
1st to 56th slice	34.90	41.26	0.00	85.45
**Four-chamber**				
1st to 30th slice	82.62	82.62	0.00	100.00
31st to 56th slice	10.35	15.42	0.00	69.23
1st to 56th slice	50.91	54.10	0.00	89.13

**Table 3:** Comparison results of 32 subjects (%).

Whole-Heart	Our Method	Larrey-Ruiz et al. [14]
1st to 30th slice—OA	**100.00**	94.42
1st to 30th slice—$A\;\left(max\right)$	**100.00**	99.81
31st to 56th slice—OA	34.28	-
1st to 56th slice—OA	73.66	**87.64**
1st to 56th slice—$A\;\left(min\right)$	**67.85**	46.95

**Table 4:** Comparison results of 20 subjects.

Whole-Heart	Our Method	Joyce et al. [28]
1st to 56th slice	**0.7833** (mIoU)	0.51 (mDice)

**Figure A1:**
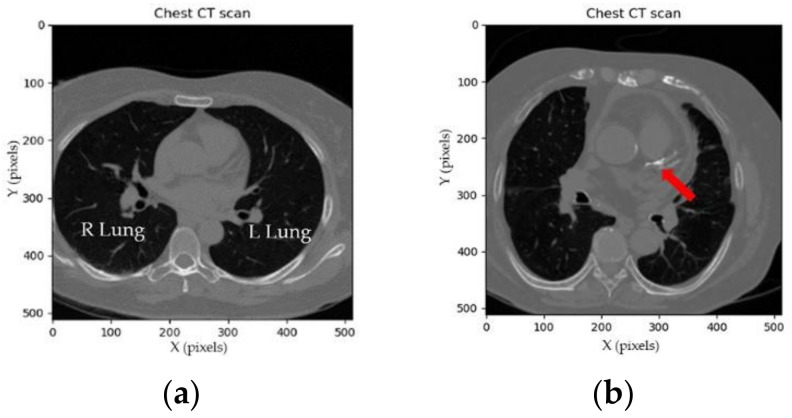
Chest contrast CT image: (**a**) normal coronary arteries; (**b**) abnormal coronary arteries consisting of calcium voxels.

**Figure A2:**
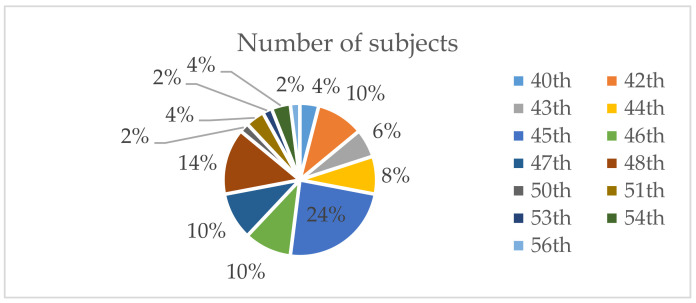
Number of subjects whose heart image ended from the 40th to 56th slice.

**Figure A3:**
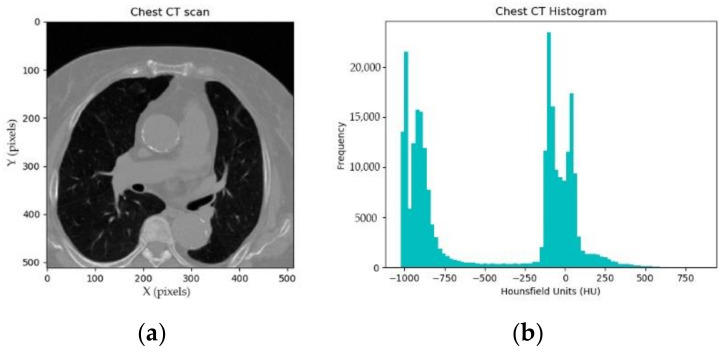
Hounsfield units in a chest CT image: (**a**) chest CT image; and (**b**) frequency histogram.

## Data Availability

No new data were created in this study. Data sharing is not applicable to this article.

## Abbreviations

The following abbreviations are used in this manuscript:

CAD	Coronary artery disease
CNN	Convolutional neural network
CT	Computer tomography
CVD	Cardiovascular diseases
DA	Descending thoracic aorta
EM	Electron microscopy
FN	False negative
FOV	Field of view
FP	False positive
GAN	Generative adversarial network
HU	Hounsfield unit
IoU	Intersection over union
LA	Left atrium
LV	Left ventricle
mIoU	Mean intersection over union
MRI	Magnetic resonance imaging
MYO	Myocardium
OA	Overall accuracy
PRI	Probabilistic rand index
RA	Right atrium
ROI	Regions of interest
RV	Right ventricle
SD	Standard deviation
TP	True positive
WHO	World health organization

## Appendix A. Chest CT Image

A computed tomography (CT) image [3,30] is a medical image that is the result of using X-ray CT scan measurements taken from different angles of a body. CT images allow the radiologist to identify disease or injury within various regions of the body without cutting. CT scans of the chest are mainly performed to gain knowledge about lung and heart anatomy, as shown in Figure A1. Figure A1a illustrates a contrast CT image in which the right lung is at the left position of the image, while the left lung is at the right position of the image. For the current study, we address a contrast CT image as a CT image. For example, the coronary CT calcium scan is used to assess CADs. A large amount of calcium pixels appearing in the coronary arteries can narrow the arteries and increase the risk of heart attack, as illustrated by the red arrow in Figure A1b.

Typically, the scanning starts from the thorax and travels to the base of lungs, known as an axial examination [38]. The number of slices per person depends on CT scanner specifications. In the current study, the CT images were scanned using a Phillips iCT 256 (described in detail in Section 4.1). Since the number of slices in which the heart appeared in the chest CT images varies from person to person, 50 subjects were randomly selected and investigated, as illustrated in Figure A2. The investigation was tracked from the first slice until the slice that contains the base of heart. Among the 50 subjects, there were 12 subjects whose heart images ended at the 45th slice.

## Appendix B. Hounsfield Unit (HU)

The Hounsfield unit (HU) [39] is a quantitative scale obtained by the linear transformation of the measurement of attenuation coefficients. Its value describes the radiodensity of a CT image and highlights different substances in the human body. The value is scaled from –1000 HU for air to +2000 HU for very dense bone and over +3000 HU for metals. The HU of substances remain the same from person to person. The HU-presenting substances of the human body in chest CT images are listed in Table A1.

**Table A1:** Hounsfield unit substances in chest CT images.

Substance	Hounsfield Unit
Air	−1000
Lung	−500
Fat	−100~−50
Water	0
Muscle	+10~+40
Liver	+40~+60
Bone	+700~+1000

Figure A3 shows a frequency of HU for one CT image. The frequency histogram describes the following information: there are more than 20,000 pixels with HU < −1000, known as air; there are more than 15,000 pixels with −1000 < HU < −500, known as lungs; there are more than 20,000 pixels with −500 < HU < +500, known as muscles, fat, heart and more; there are a few pixels with HU > +500, known as bone.

## Appendix C. Algorithms

The following algorithms were used in this manuscript.

**Table A2:** Generation process of a convex hull of foreground mask.

Algorithm 1: Generate a convex hull of foreground mask
Input: Whole chest CT image in grayscale format (512 × 512)
Output: Convex hull of foreground mask in binary format (512 × 512)
1: Begin
2: centroid_clusters = KMean(input_image, n_cluster = 2)
3: fg_threshold = mean(centroid_clusters)
4: if input_image > fg_threshold:
5: fg_mask = 1
6: else
7: fg_mask = 0
8: fg_mask = morphology.opening(fg_mask, n_iteration = 2)
9: ch_fg_mask = morphology.convex_hull(fg_mask)
10: End

**Table A3:** Generation process of a lung mask, a convex hull of lung mask and an intermediate heart mask.

Algorithm 2: Generate a lung mask, a convex hull of lung mask and an intermediate heart mask
Input: Whole chest CT image in grayscale format (512 × 512); Convex hull of foreground mask in binary format (512 × 512)
Output: Intermediate heart mask in binary format (512 × 512)
1: Begin
2: foreach pixel in input_image:
3: if ch_fg_mask == 0:
4: fg_pixel = 255
5: else
6: fg_pixel = pixel
7: if fg_pixel < fg_threshold:
8: lung_mask = 1
9: else
10: lung_mask = 0
11: lung_mask = morphology.closing(lung_mask, n_iteration = 2)
12: ch_lung_mask = morphology.convex_hull(lung_mask)
13: int_heart_mask = ch_lung_mask AND invert(lung_mask)
14: End

**Table A4:** Generation process of a spine mask, a convex hull of spine mask and a heart mask.

Algorithm 3: Generate a spine mask, a convex hull of spine mask and a heart mask
Input: Whole chest CT image in grayscale format (512 × 512); Intermediate heart mask in binary format (512 × 512)
Output: Heart mask in binary format (512 × 512)
1: Begin
2: foreach pixel in input_image:
3: if int_heart_mask == 0:
4: int_heart_pixel = 0
5: else
6: int_heart_pixel = pixel
7: centroid_clusters = KMean(int_heart_pixel, n_cluster = 3)
8: spine_threshold = max(centroid_clusters)
9: if int_heart_pixel > spine_threshold:
10: spine_mask = 1
11: else
12: spine_mask = 0
13: spine_mask = morphology.closing(spine_mask, n_iteration = 2) 14: spine_mask = morphology.opening(spine_mask, n_iteration = 2) 15: (x0,y0) = measure.regionprob(spine_mask) 16: (xc,yc) = measure.regionprob(spine_mask)
17: ch_spine_mask = spine_mask[y0 : , : xc] is set to 1
18: ch_spine_mask = ch_spine_mask[yc : , : ] is set to 1
19: heart_mask = int_heart_mask AND invert(ch_spine_mask)
20: End

**Table A5:** Generation process of segmented heart pixels.

Algorithm 4: Segment a heart image
Input: Whole chest CT image in HU format (512 × 512); Heart mask in binary format (512 × 512)
Output: Segmented heart pixel in HU format (512 × 512)
1: Begin
2: foreach pixel in input_image:
3: if heart_mask == 0:
4: heart_pixel = −1000
5: else
6: heart_pixel = pixel
7: End

**Table A6:** Generation process of a refined convex hull of lung mask.

Algorithm 5: Refine a convex hull of lung mask
Input: Lung mask in binary format (512 × 512 × 56)
Output: Refined convex hull of lung mask in binary format (512 × 512 × 56)
1: Begin
2: for n in range(56): 3: if y0 of lung_mask [n] > y0 of lung_mask [n-i]:
4: ch_lung_mask [n] = ch_lung_mask [n] OR ch_lung_mask [n-i]
5: End
